# A Spectroscopic Study of the Insulator–Metal Transition in Liquid Hydrogen and Deuterium

**DOI:** 10.1002/advs.201901668

**Published:** 2019-11-27

**Authors:** Shuqing Jiang, Nicholas Holtgrewe, Zachary M. Geballe, Sergey S. Lobanov, Mohammad F. Mahmood, R. Stewart McWilliams, Alexander F. Goncharov

**Affiliations:** ^1^ Key Laboratory of Materials Physics Institute of Solid State Physics Chinese Academy of Sciences Hefei Anhui 230031 China; ^2^ Geophysical Laboratory Carnegie Institution of Washington Washington DC 20015 USA; ^3^ Department of Mathematics Howard University 2400 Sixth Street NW Washington DC 20059 USA; ^4^ GFZ German Research Center for Geosciences Section 3.6, Telegrafenberg 14473 Potsdam Germany; ^5^ School of Physics and Astronomy and Centre for Science at Extreme Conditions University of Edinburgh Edinburgh EH9 3FD UK; ^6^ Present address: US Food and Drug Administration 645 S Newstead Ave. St. Louis MO 63110 USA

**Keywords:** extreme pressure and temperature, hydrogen, insulator‐to‐metal transition

## Abstract

The insulator‐to‐metal transition in dense fluid hydrogen is an essential phenomenon in the study of gas giant planetary interiors and the physical and chemical behavior of highly compressed condensed matter. Using direct fast laser spectroscopy techniques to probe hydrogen and deuterium precompressed in a diamond anvil cell and laser heated on microsecond timescales, an onset of metal‐like reflectance is observed in the visible spectral range at *P* >150 GPa and *T* ≥ 3000 K. The reflectance increases rapidly with decreasing photon energy indicating free‐electron metallic behavior with a plasma edge in the visible spectral range at high temperatures. The reflectance spectra also suggest much longer electronic collision time (≥1 fs) than previously inferred, implying that metallic hydrogen at the conditions studied is not in the regime of saturated conductivity (Mott–Ioffe–Regel limit). The results confirm the existence of a semiconducting intermediate fluid hydrogen state en route to metallization.

## Graphical abstract

By applying microsecond single‐ to several‐pulse laser heating in a diamond anvil cell in combination with pulsed broadband laser probing detected via a streak camera, the onset of metallic optical reflectance is determined in fluid hydrogen and deuterium above 3500 (1000) K at 150–172 GPa.

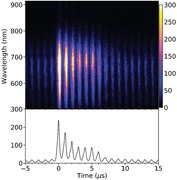

## 1 Introduction

The insulator‐to‐metal transition (IMT) in hydrogen is one the most fundamental problems in condensed matter physics.^1^ In spite of seeming simplicity of hydrogen (2p + 2e in the molecule), the behavior of this system at high compression remains poorly understood. The structural, chemical, and electronic properties of hydrogen and other molecular system are strongly dependent on pressure (density); at high pressures the relative stability of atomic over molecular configurations increases due to an increase in the electronic kinetic energy thus easing the transformation to a metallic state.^2^, ^3^ Principal challenges include understanding the intermediate paired, mixed, and monatomic states, both solid and fluid;^3^, ^4^ the mechanism and pressure–temperature (*P–T*) conditions of IMT; the location of critical and triple points related to a change in the transition character and implications to high‐temperature superconductivity and the internal structure, composition, temperature, and magnetic fields of gas giant planets.^5^, ^6^, ^7^ Currently, the IMT in hydrogen is expected to occur in two regimes: at low *T* (<600 K) in the dense solid, where quantum effects are expected to dominate and at high temperatures in the fluid state, where classical entropy must play an important role. In the former scenario there is a possibility of quantum melting where solid H_2_ liquefies into a metallic quantum fluid.^8^, ^9^ However, recent investigations found solid hydrogen at low temperatures (<200 K) transforming to a conducting state with a narrow or zero bandgap above 360 GPa,^10^, ^11^ making uncertain the existence of a ground‐state metallic fluid in this regime. The nature of the metallic fluid at high temperatures, as relevant to planetary interiors, also remains to be established, with questions persisting about electronic transport properties such as electrical conductivity^12^, ^13^, ^14^, ^15^, ^16^, ^17^, ^18^ and the related chemical state.^18^, ^19^

The IMT in fluid hydrogen was initially predicted as the first‐order transition ending in a critical point at very high temperatures (10–17 kK).^5^, ^6^, ^7^ However, dynamic gas gun and laser driven experiments probing changes in electrical conductivity and optical reflectance found a continuous transition to a metallic state at 50–140 GPa^12^, ^20^, ^21^ at lower temperatures, implying a critical temperature below 3 kK. First‐principles theoretical calculations suggest values of ≈2 kK but yield very different critical pressures, and correspondingly positions of the transformation line.^22^, ^23^, ^24^, ^25^, ^26^ Arguably, the coupled electron‐ion Monte Carlo calculations^19^ provide the most accurate predictions; they suggest that the dissociation and metallization transitions coincide (cf. ref. 18), and the critical point is located near 80–170 GPa and 1600–3000 K. While dynamic compression experiments, which explore a variety of *P–T* pathways, agree on existence of the metallic states detected via electrical, optical, and density measurements,^12^, ^14^, ^15^, ^20^, ^21^, ^27^ lower temperature data show inconsistent results on the position of metallization and the optical character of intermediate states.^14^, ^15^

Static diamond anvil cell (DAC) experiments combined with laser heating probing similar low temperature fluid states have also yielded controversial results on the electronic properties of hydrogen and the location of the phase lines.^13^, ^28^, ^29^, ^30^, ^31^, ^32^ The difficulty of interpreting these optical DAC experiments is due to indirect probing of the state of hydrogen,^30^, ^31^ or detection of reflectance signals superimposed with those of other materials in the DAC cavity and interpreted assuming a priori a direct transformation from insulator to metal.^13^, ^29^, ^32^ The latter results, reporting transient reflectance and transmission at a few laser wavelengths, have been found inconsistent with the proposed IMT, while an indirect transformation via intermediate‐conductivity states is a plausible alternative.^12^, ^14^, ^15^, ^28^, ^33^, ^34^ One of the major drawbacks of the majority of preceding dynamic and static experiments is an extreme paucity of robust spectroscopic observations, which are critical for assessing the material electronic properties.

Here, we address the challenges raised above by exploring experimentally the electronic states of hydrogen and deuterium in the *P–T* range where the IMT was previously reported but not sufficiently characterized. To overcome the challenges in sustaining hydrogen at these conditions and probing it spectroscopically we applied microsecond single‐ to several‐pulse laser heating in combination with pulsed broadband‐laser probing (Figure S1, Supporting Information). We show that the transition in *P–T* space includes several stages where hydrogen transforms from a transparent insulating state, to an optically absorptive narrow‐gap semiconducting state, and finally to a metallic state of high reflectance. The metallic state exhibits a plasma edge in the visible spectral range, implying a plasma frequency and electronic scattering time that contrasts with previous inferences,^14^, ^20^, ^21^ mainly based on the Mott–Ioffe–Regel (MIR) limit approximation in which the electronic mean‐free‐path reaches the interatomic spacing, and in stark disagreement with the prior static experiments probing hydrogen at few laser wavelengths.^13^

## 2 Experimental Section

A strong extinction of the transmitted light was detected when hydrogen was laser heated above a certain threshold laser heating power (Figure S2, Supporting Information). The transient absorption reaches a maximum shortly after the arrival of the heating pulse, followed by a regaining of the transmitted signal. The absorption spectra, measurable only at lower temperatures where transmission remains detectable, consistently show an increased transparency toward lower energies similar to that reported previously for absorptive fluid hydrogen, suggesting that hydrogen in this regime is semiconductor‐like with a bandgap of the order of ≈1 eV.^28^ Transient reflectance signal in this regime shows a small, spectrally independent increase (Figure S3, Supporting Information) which can be explained by a small change in the refractive index of H_2_ (D_2_) correlated with bandgap reduction.^20^ In this regime, peak temperature measured radiometrically (Figure S4, Supporting Information) tends to increase slowly with laser power, while the duration at which the sample remains hot (and thus emits) increases^28^, ^29^, ^33^ (Figure S5, Supporting Information); temperature increases more rapidly at higher laser power.

**Figure 1:**
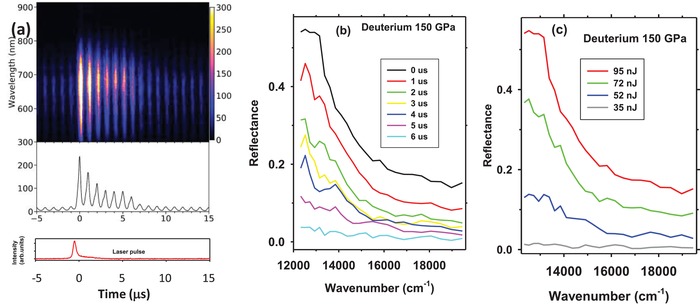
Transient optical reflectance data of deuterium at 150 GPa. a) Spectrogram of time dependent reflectance of laser heated deuterium at 150 GPa. The signal intensity is color‐coded as shown in the bar attached to the right side of the spectrogram. The integrated in wavelength signal is shown in the middle panel, while the heating pulse is shown in the bottom panel. b) The transient reflectance spectra measured at the different times after the arrival of the heating pulse (see the legend), probing the sample at varying temperature. c) Maximum reflectance as a function of laser pulse energy (see the legend). Temperature is 4400 (600) K at peak heating in (b).

At temperatures exceeding 3000 K, a strong transient reflectance signal from hydrogens was detected in all samples studied (**Figure** **1**; Figures S6 and S7, Supporting Information). Reflectance of hot transformed hydrogen isotopes (H and D) exceeds the background reflectance substantially and is characterized by a spectrally variable magnitude. At the conditions where the reflective hydrogen forms, it has a sufficiently large emissivity so its thermal radiation can be reliably collected and spectrally analyzed, enabling direct determination of the sample temperature (Figure S4, Supporting Information).^28^ The reflectance spectra (Figure 1) show a large increase to lower energy, a characteristic of metals. Within a single heating event, the reflectance reaches a maximum when the highest temperature is reached (just after heating pulse arrival) and diminishes as the sample cools (Figure 1b). The overall reflectance value increases with the laser heating pulse energy (and hence the maximum sample temperature) (Figure 1c). These transient changes at high temperatures are reversible (Figure 1a), sometimes occurring with relatively smaller changes to the background attributed to laser absorber movement; thus, they must manifest a transition in the state of hydrogens at these extreme *P–T* conditions. As in our previous work,^28^ Raman spectra measured before and after heating to the presently achieved conditions showed the vibron mode of hydrogen and do not show any extra peaks that could be related to irreversible chemical transformations that would occur as a result of exposure of hydrogen to extreme *P–T* conditions.

**Figure 2:**
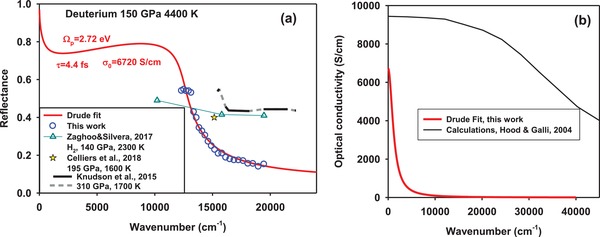
Optical conductivity of metallic deuterium. a) Reflectance spectrum of deuterium; the experimental data (circles) are compared to the Drude fit (see Supporting Information). Shown for comparison, albeit at different thermodynamic conditions, are results of DAC experiments for hydrogen^13^ (triangles) using three monochromatic laser probes, and laser driven dynamic experiments for deuterium that used passive spectroscopy^15^ (solid black lines with dashed gray interpolation) and monochromatic probing (star).^14^ b) Optical conductivity from this work compared to that theoretically computed in ninefold compressed deuterium at 3000 K.^17^

The reflectance measurements of hydrogen all yielded qualitatively similar spectra (Figure S7, Supporting Information) with the pronounced increase in intensity toward low energy. These spectra can be fitted with a variety of different models, but it is found that a Drude free electron model (Supporting Information), which employs the plasma frequency *Ω*_P_ and the mean free time between the electron collisions τ as the free parameters, fits the data well, yielding *Ω*_P_ = 2.72(5) eV and *τ* = 4.4(1.6) fs for deuterium, where the detected reflectance was largest (**Figure** **2**). In these calculations, it is assumed that the refractive index of warm nonmetallic hydrogen in contact with metallic hydrogen is 3.0 at extreme *P–T* conditions following recent dynamic compression measurements.^14^ Furthermore, our reflectance data in the high frequency limit can only be accurately fitted by including a bound electron contribution to the electronic permittivity function of metallic hydrogen (ε_b_ = 3.1 for the representative case above). The uncertainty of our estimation of the DC conductivity σ_DC_ = *Ω*_P_^2^τ is of the order of 30%, σ_DC_ = 6700(2400) S cm^−1^.

**Figure 3:**
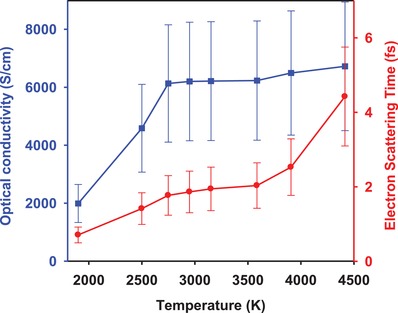
Optical conductivity and electron scattering time through metallization of deuterium at 150 GPa. The results are based on Drude fitting of sequential spectra measured during cooling from the peak temperature in a single experiment. Temperatures below 2700 K are determined via linear extrapolation with time. Values not corrected for deviation from Drude behavior at lower temperatures (Figure 4).

**Figure 4:**
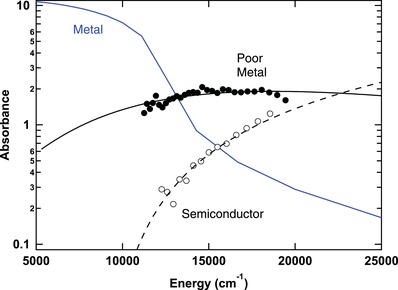
Absorption spectra through metallization at 140–150 GPa. Spectrum of metallic deuterium at 150 GPa, 3000 K determined from Drude fit to reflectance and having σ_DC_ = 6700 S cm^−1^, τ = 4.4 fs is the solid blue line (after Figure 2). Measured spectrum of a transitional, poor‐metal deuterium state at 150 GPa, *T* < 2700 K, given by the filled circles (red points in Figure S2c, Supporting Information), is best fit by a Smith–Drude model (solid black line), here with σ_DC_ = 35 S cm^−1^, τ = 0.3 fs, *C* = −0.95. Prior data on semiconducting hydrogen^28^ at 141 GPa and 2400 K (open symbols) are best fit by a Tauc model (dashed black line) with gap energy of ≈1 eV, corresponding to σ_DC_ ≈ 15 S cm^−1^, τ ≈ 0.03 fs. A 1 µm thick layer is assumed in calculations.

The reflectance spectra (Figure 1b,c) at various temperatures varied either during cooling down or by changing the laser heating energy can be also fit with the Drude model. The results of time domain experiments on cooling down show a change in a slope in the Drude parameters at the critical onset temperature *T*_c_ (**Figure** **3**), where the reflectance becomes less than approximately 10%. The most prominent change is in the DC conductivity, which is almost constant above the onset transition (although the reflectance values vary) and start dropping down fast below T_c_ manifesting the transition. This is qualitatively similar to the recently reported behavior of deuterium under ramp compression near 200 GPa, albeit probed as a function of pressure.^14^ It is also found that *τ* decreases from the metallic state through the transition (Figure 3). Furthermore, it is found that the electronic permittivity ε_b_ increases; although the plasma frequency increases in the metallic state, the “screened” plasma frequency *Ω*_P_/$\sqrt{\varepsilon_{b}}$ remains constant and drops in a semiconducting state (Figure S8, Supporting Information). These observations suggest an electronic oscillator frequency shift from high energy toward zero as metallization progresses, which is further supported by our optical absorption data (**Figure** **4**), in the regime of low reflectance. This is a common feature in insulators undergoing metallization (e.g., ref. 35) resulting from charges becoming increasingly less bound, while the scattering time also increases considerably into the metallic state, which can also be attributed to a transformation from localized to delocalized carriers.

## 3 Discussion

**Figure 5:**
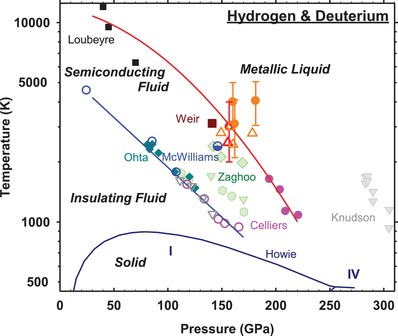
Phase diagram of hydrogen at extreme *P–T* conditions. Filled orange and filled crossed red circles indicate conditions of the metallic state detected via optical reflectance in this study for hydrogen (H) and deuterium (D), respectively. The large error bars (nearly 1000 K) are due to low sample emissivity and temperature gradients. Open orange and red upward triangles correspond to *P–T* conditions where H and D reflectance respectively was lower than a few percent and our Drude analysis shows a sharp decline in the DC conductivity (Figure 3). A thermal pressure of 2.5 GPa/1000 K is included.^28^ Open and half open blue circles are conditions in H where the onset of absorption occurs and a semiconducting state (≈0.9 eV bandgap) was detected, respectively, directly measured using a similar DAC technique as in this work.^28^ Open and filled pink circles (light gray triangles) are the results of gradual laser compression at NIF^14^ (Z‐machine^15^) corresponding to reaching the absorptive and reflecting D states, respectively. Solid brown square is the result of reverberating shock experiments detecting metallization of H by electrical conductivity measurements.^12^, ^15^ Filled black squares are IMT measured in single‐shock experiments in precompressed samples; no major differences between D and H were indicated.^21^ The results of DAC optical experiments reported as an abrupt insulator–metal transition are shown by light green hexagons and triangle for H and light green diamonds for D.^13^, ^29^, ^32^ Solid cyan diamonds are DAC experiments in H showing change in temperature versus heating power dependence interpreted as phase transformation to a metal.^30^, ^31^ Solid red and blue line through the data are the suggested phase boundaries for semiconducting and metallic hydrogen. The melting curve and solid‐state boundaries are from ref. 41.

The DC (electrical) conductivities inferred here are in reasonable agreement with the results of theoretical calculations (≈10 000 S cm^−1^)^16^, ^17^, ^19^, ^36^, ^37^, ^38^ and compares well to the dynamic experiments on metallic hydrogen and deuterium.^12^, ^14^ However, our experiments suggest a more than an order of magnitude longer electronic collision time τ in the metallic state implying that the conducting electrons in hydrogens at the conditions studied are not in the MIR limit. In the absence of the spectral reflectance data, the validity of the MIR conditions was a common assumption in analyzing the dynamic compression data;^14^, ^20^, ^21^ theoretical calculations were in a general agreement predicting a very damped Drude response^17^, ^39^ (Figure 2b). Our reflectance spectra are in partial disagreement with those reported in the dynamic experiments^15^ (Figure 2a), though these refer to substantially different *P–T* conditions (**Figure** **5**), do not cover a near IR spectral range, are obtained on a hydrogen‐LiF interface, and use a passive spectroscopy technique sensitive to diffuse scattering. This makes a direct comparison of reflectance spectra possibly inappropriate, however, some evidence of a sharper rising reflectance to lower energy, similar to that observed here, is noted in these data. Recent DAC experiments^13^ at similar conditions to the present results report a value of σ_DC_ = 11 000 S cm^−1^, which agrees broadly with our determination, but the reflectance results differ drastically (Figure 2a): a Drude fit to those data^13^ yielded a larger plasma frequency (*Ω*_P_ = 20.4 eV) and smaller electron collision time (τ = 0.13 fs) compared to our results. The distinction between our results and those of ref. 13 are unlikely due to a difference in the probed *P–T* conditions. In fact, we find the onset of metallic conditions at higher temperature than in refs. 13, 29, in better agreement with the results of dynamic experiments^12^, ^14^, ^15^ (Figure 5) with regard to the *P–T* conditions of metallization. The differences in inferred metallization conditions and spectral response may be due to the larger background signal in refs. 13, 29, from a tungsten layer in the probed sample region, the optical properties of which at extreme *P–T* conditions are unknown.

The sharp reflectance rise in the visible spectral range documented here is remarkable. We assign it to the presence of a plasma edge common for many metals, for example gold and silver. Such electronic excitations with the frequencies near the plasma edge are not unusual for simple metals; these would represent the electronic transitions to excited bound states, which could correspond to weakly bound dimers of hydrogens. In this regard, we have attempted to reproduce our reflectance spectrum by using a two‐oscillator model (Figure S9, Supporting Information). However, the DC conductivity in this model must be near σ_DC_ = 61 000 S cm^−1^, an order of magnitude larger than for the Drude model, which is inconsistent with the dynamic electrical conductivity experiments.^12^

The results presented here clearly demonstrate the existence of two transformation boundaries corresponding to the formation of absorptive and reflecting hydrogen (Figure 5). The one at lower *P–T* conditions has been established in dynamic^14^, ^15^ and DAC experiments.^28^, ^30^, ^31^, ^33^ It has been suggested that this boundary is related to a bandgap closure,^14^, ^15^, ^28^, ^34^ rather than the plasma transition. However, the absorption edge is broad (≈1 eV),^28^ while the transition is rather abrupt (a few hundreds of degrees); such large temperature driven bandgap changes are normally uncommon. This semiconducting state occupies a large *P–T* space (Figure 5),^28^ while the new data suggest a rather abrupt metallization at higher *P* and *T*. The bandgap closure is usually treated as a pressure (density) driven transformation, while both the previous absorption and the present reflectance results indicate a strongly temperature driven transition (see also refs. 28, 31). This suggests that the observed phenomena are related to the molecular instability and the observed boundary corresponds to a temperature driven partial molecular dissociation. Near the boundary at approximately 150 GPa, the molecular binding energy is approximately equal to the zero point energy.^3^, ^40^ In this interpretation, upon increasing the temperature, molecules first begin to dissociate and recombine frequently, producing a state with a measurable electrical and optical conductivity.^12^, ^14^, ^28^ It is not a metallic state, as the charge carriers are mostly localized. To reach the metallic state one needs to dissociate a critical fraction of molecules (e.g., 40%^17^) and enable nonlocal carrier transport, which occurs at higher *P–T* conditions. We note in this regard that semimetallic solid hydrogen state has been recently reported^10^ at low temperature and higher pressure; however, the nature of that state is likely different emerging from the topology of the electronic band structure.

Our data using direct temperature measurements show a reasonably good agreement with the results of dynamic experiments,^12^, ^14^, ^15^, ^21^, ^27^ the majority of which is based on calculated temperature. Given this good consistency especially with the most recent calculated temperatures for dynamic compression experiments,^14^, ^15^ including updated calculations for ref. 12, our results suggest the basic accuracy of those calculations. DAC results reported by the Harvard group (green symbols) suggest a transition at ≈1000 K lower temperatures (Figure 5). Our results do not suggest any major isotope effect (cf. ref. 32), which is consistent with previous shock wave results.^12^, ^21^ The lines of conductance and metallization become closer in *T* at higher *P* (Figure S10, Supporting Information) as expected on approaching a critical point, however the data suggest that they both would intersect the melting line first. The pressure range of 170 – 250 GPa at temperatures just above the melting line can be expected to be anomalous. This *P–T* space has been probed in two recent high‐temperature Raman experiments.^41^, ^42^ It is interesting that Zha et al.^42^ detected an anomaly in the pressure dependence of the liquid hydrogen vibron band at 140–230 GPa, which can be related to the presence of conducting mixed molecular‐atomic fluid hydrogen. However, they find that fluid hydrogen remains molecular at 300 GPa, which calls for improved *P–T* metrology in dynamic laser and resistively driven static experiments (Figure S10, Supporting Information).

## 4 Conclusions

Our spectroscopic investigation of fluid hydrogens in the regime of molecular dissociation and metallization showed the complexity of the phenomena suggesting a two‐stage transition with a semiconducting intermediate state preceding that of a free‐electron metal. The reflectance spectra of the metallic hydrogens show the presence of a plasma edge, which allow constraining the electronic conductivity parameters. We find an electronic relaxation time that is much larger than previously inferred, suggesting that electronic transport is not in the MIR saturation regime as previously thought.

## Conflict of Interest

The authors declare no conflict of interest.

## Supporting information

Supporting Information
