# Supplementary material for: A Spectroscopic Study of the Insulator–Metal Transition in Liquid Hydrogen and Deuterium
Source: Adv Sci (Weinh). 2019 Nov 27;7(2):1901668. doi: 10.1002/advs.201901668 (PMC6974937; doi:10.1002/advs.201901668)
Supplement: Supplementary file 1 — Supporting Information [file ADVS-7-1901668-s001.pdf]

Copyright WILEY-VCH Verlag GmbH & Co. KGaA, 69469 Weinheim, Germany, 2019.

Supporting Information

for *Adv. Sci.,* DOI: 10.1002/advs.201901668

A Spectroscopic Study of the Insulator–Metal Transition in Liquid Hydrogen and Deuterium

*Shuqing Jiang, Nicholas Holtgrewe, Zachary M. Geballe, Sergey S. Lobanov, Mohammad F. Mahmood, R. Stewart McWilliams, and Alexander F. Goncharov**

Supplemental information for

A spectroscopic study of the insulator-metal transition in liquid hydrogen and deuterium

Shuqing Jiang[1,2], Nicholas Holtgrewe[2,3,]†, Zachary M. Geballe[2], Sergey S. Lobanov[2,4]‡,

Mohammad F. Mahmood[3], R. Stewart McWilliams[5], Alexander F. Goncharov[1,2]*

Alexander Goncharov
E-mail: agoncharov@carnegiescience.edu

This PDF file includes:
Methods (2 pages)
Table 1.
Figs. S1 to S10
References for SI reference citations

Methods

Sample preparation. We investigated three H_2 and one D_2 samples gas loaded in the DAC with beveled diamonds of 40-70 μm diameter central culets and compressed to pressures of 120-172 GPa (Table 1). Alumina coatings of a submicrometer thicknesses have been sputtered on the diamond anvil prior the gas loadings. Thin Ir or boron-doped diamond (the latter of approximately 1 μm thick) plates of 20-40 μm linear dimensions with or without small (<10 μm in diameter) cylindrical holes were positioned in the high-pressure cavity to serve as the laser radiation absorbers.

Dynamic laser heating and optical probes in the diamond anvil cell. Our time-resolved single pulse laser heating diamond anvil cell experiments combine measurements of optical emission, transmission and reflectance spectroscopy in the visible spectral range (480-750 nm) using a streak-camera, as has been described in our previous publications [1, 2] (*SI Appendix Fig. S1*); we used a grating with 75 gr/mm covering the overall visible spectral range (*SI Appendix Fig. S1*), which eliminates the necessity to stitch spectra measured at different spectral positions. The laser pulses of 4–10 μs duration are sufficiently long to transfer heat to the hydrogen sample in the hole of the heat absorbers creating a localized heated state of several μm in linear dimensions and a few μs long as determined in our FE calculations [2]. The optical spectroscopic probes, aligned to the heated spot, were used in a confocal geometry suppressing spurious probe signals. Transient transmittance and reflectance were obtained using a pulsed broadband supercontinuum (SC, 1 MHz, 1 ns, 480–720 nm) probe having focal spots of approximately 6 μm in diameter (*SI Appendix Fig. S1*) that is spatially filtered with a confocal aperture of some 50% larger in diameter.

Time resolved (with the resolution down to 0.5 μs) sample temperature was obtained from fitting thermal radiation spectra emitted by the coupler and hot sample to a Planck function (*SI Appendix Fig. S4*). These were normally determined in a separate experiment with identical heating without probing, and due to weak thermal radiation were in some cases integrated over a number of laser heating events (5-20) to improve signal-to-noise. The measured temperature should be treated cautiously as the thermal radiation measured represent a sum of contributions from the coupler and the sample. Commonly, the heat absorber has higher temperature than the sample and in the case of Ir coupler emits more because of difference in emissivity, while boron doped diamond emits very little. However, the sample emissivity changes substantially once it becomes absorptive, suggesting that the measured thermal emission in this regime characterizes the sample temperature. Additionally, FE calculations [1-3] have been used to model the temperature distribution in the high-pressure cavity.

Pressure was determined at room temperature using the spectral position of the Raman vibron peaks (*e.g.* Ref. [4]) and additionally the stressed diamond edge before and after the laser heating experiments (Table 1). Pressure was found to remain essentially constant (within <3 GPa) between heating cycles in the case diamonds remained intact. Pressure drops (or total failures) in some of these experiments was due to cracks in the diamond anvils, which develop shortly after the laser shots. A thermal pressure of 2.5 GPa/1000 K was assumed to estimate the pressure conditions at high temperature[2]. At each desired pressure, temperature was increased

stepwise with an increase of the heating laser power. This was controlled by rotating λ/2 wave plate coupled to a cube polarizing beamsplitter.

Transient optical data reduction. To determine the transient reflectance spectra of conducting hydrogen at extreme *P-T* conditions we measured the reflectance of the outside diamond-air interface as a natural reflectance standard. The background Fresnel reflectance from the diamond-alumina and alumina-hydrogen interfaces were subtracted (*SI Appendix Figs. S3, S6*). No correction has been made for the attenuation made by an absorptive conducting deuterium (cf. Ref. [5]) due to the optimal experimental condition achieved here where the hot metallic sample is confined within a thin layer between absorber and alumina coating. The reflectance spectra of hydrogen, the samples of which were not positioned optimally, have been used only for the qualitative analysis. The use of the diamond anvil coating improves the sample insulation and makes thinner the absorptive semiconducting sample within the sample cavity, which screens the reflection from the metallic state.

Drude model. Reflectance spectra are well-fitted by a Drude model having conductivity of the form $\sigma=\sigma_0(1-i\omega\tau)^{-1}$ where ω is the angular frequency. The DC conductivity is $\sigma_0=\Omega_p^2\tau$, in which Ω_p is the plasma frequency, τ the scattering time, and ε_0 the permittivity of free space. The dielectric constant is $\varepsilon^* = \varepsilon_b+i\sigma/\omega$where ε_b is the bound electron contribution to the dielectric constant. A range of ε_b from 1 to n_H^2 (n_H =3) are examined when assessing uncertainty; we generally found ε_b=3.1 provided a better fit to the data. The corresponding index of refraction is $n^* = \sqrt{\varepsilon^*}$. The absorption coefficient is determined as 2ω*Im(n^*)/c. The reflectance of the interface between the metallic and semiconducting hydrogen is modelled as $R = |(n^*-n_H)/(n^*+n_H)|^2$, where the refractive index of the semiconducting state (n=3.0) is taken from Ref. [6], where it was measured in very similar thermodynamic conditions in laser driven ramp compression experiments. The Smith-Drude model has been used following the formulations in Refs. [2, 6], i.e. $\sigma=\sigma_0(1-i\omega\tau)^{-1}[1+C(1-i\omega\tau)^{-1}](1+C)^{-1}$.

Table 1. Information on the experimental conditions and observables.

#	Material	P before (GPa)	P after (GPa)	Absorption	Reflectance	Coupler	Al2O3 coating
1	Deuterium	124	124	Yes	No	B:C	Yes
1	Deuterium	150	130	Yes	Yes	B:C	Yes
2	Hydrogen	146	149	Yes	No	Ir	No
2	Hydrogen	155	failure	Yes	Yes	Ir	No
3	Hydrogen	151	failure	Yes	Yes	B:C	Yes
4	Hydrogen	172	157	Yes	Yes	B:C	Yes

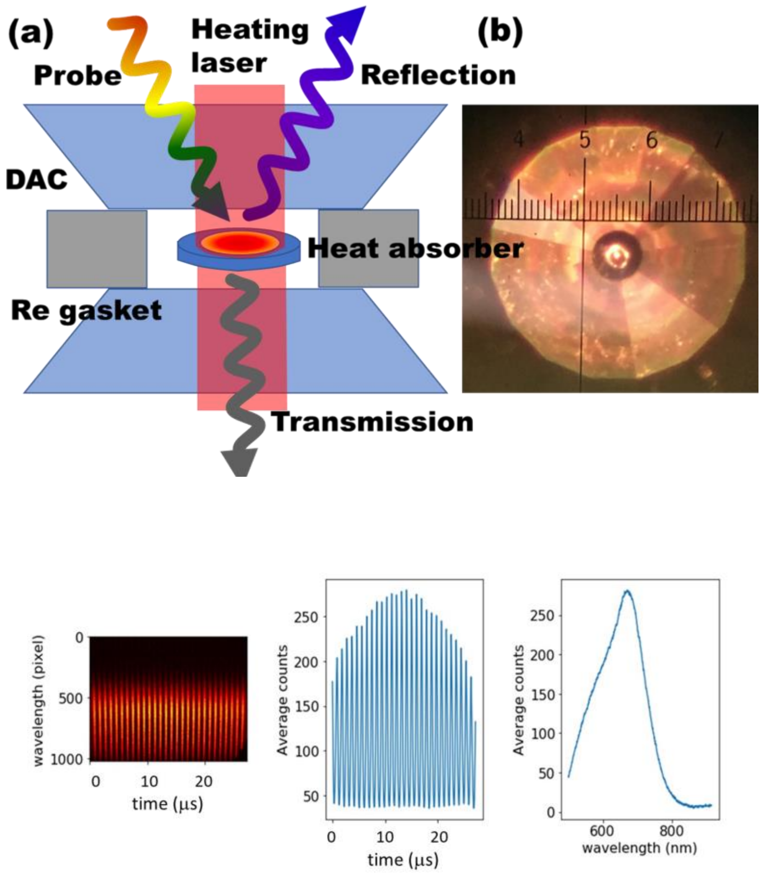

Figure S1. Experimental geometry of a diamond anvil cell experiment along with an experimental schematic (left) and microphotograph (right) in the top and characteristics of a spectral probe (supercontinuum) laser measured via a streak camera: spectrogram (left) and integrated in wavelength (center) and time (right) in the bottom.

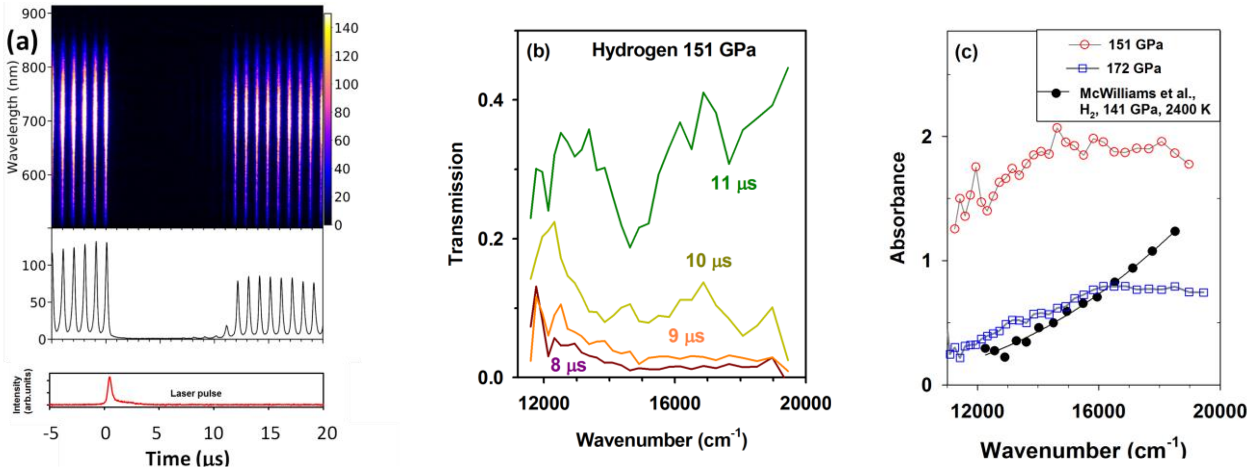

Figure S2. Transient optical transmission data of hydrogen at 151 and 172 GPa. Left panel (a): transmission spectrogram at 151 GPa, which shows the time dependence of the pulsed supercontinuum (SC) signal transmitted through the sample during pulsed laser heating (bottom panel). The signal is dispersed by a diffraction grating and recorded via the streak camera. The signal intensity is color-coded as shown in the bar attached to the right side of the spectrogram. The SC pulses are arriving with a 1 μs time interval. The laser heating pulse shown in the bottom arrives after the SC laser pulse at the 0th μs. The temperature was too low (<3000 K) to be detected in this single event experiment. The middle panel (b) shows the transmission spectra at different times (labeled near the curves) after the arrival of the heating pulse and the right panel (c) depicts the optical absorption spectra at 151 and 172 GPa (on cooling) in comparison to that reported in Ref. [2].

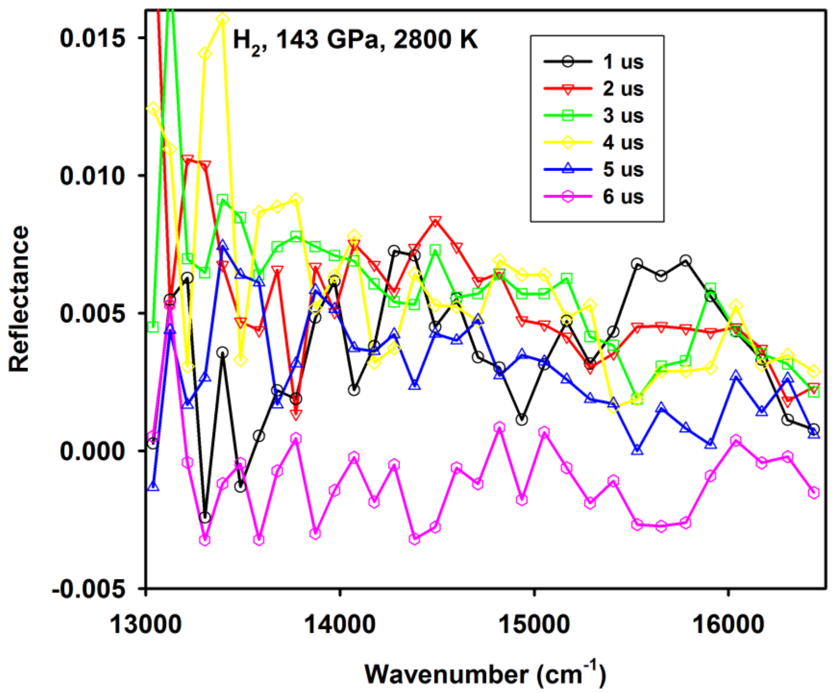

Figure S3. Reflectance spectra at 143 GPa. The heating pulse that arrives near the 0th microsecond causes a small uniform increase of reflectance. Each spectrum measured every 1 μs using a single SC pulse is shown at the different times after the arrival of the heating pulse (see the legend). The spectra are normalized to the reflectance of the diamond-air interface (the anvil table) and the background signal was subtracted.

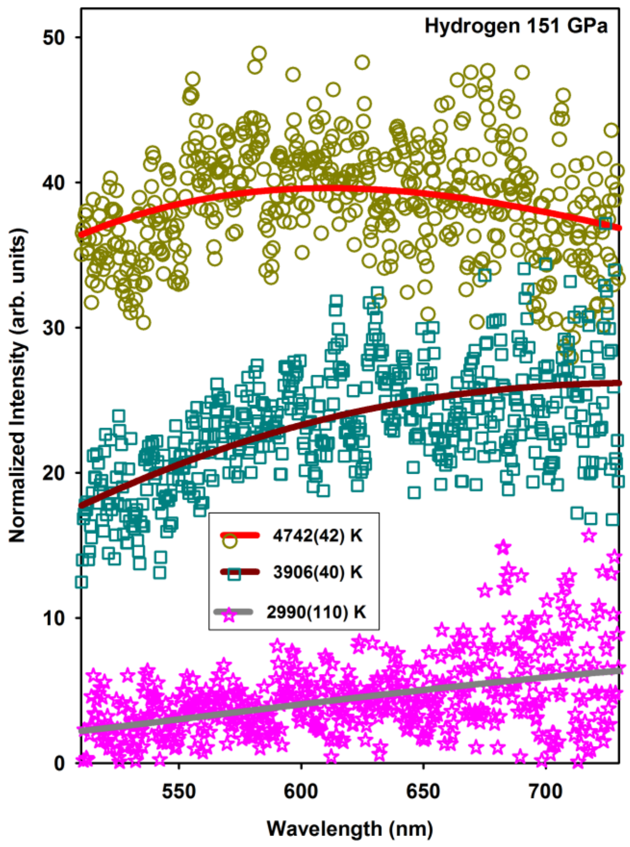

Figure S4. Spectroradiometric transient temperature measurements for hydrogen at 151 GPa. The data for various representative times are obtained in the course of Heat 5 (Fig. S5).

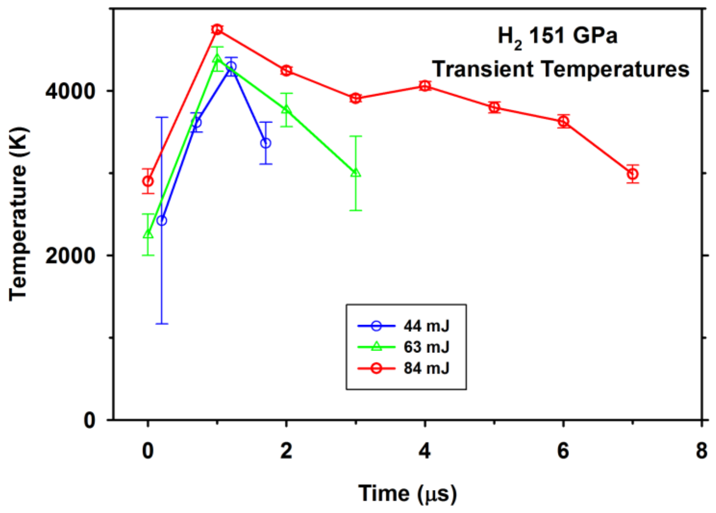

Fig. S5. Transient temperature measured with spectroradiometry at 151 GPa with hydrogen as a sample. Reflectance measurements showed a very small increase for Heats 3 (44 mJ) and 4 (63 mJ), while a stronger reflectance with a characteristic increase toward the lower energy side was detected in the course of Heat 5 (84 mJ).

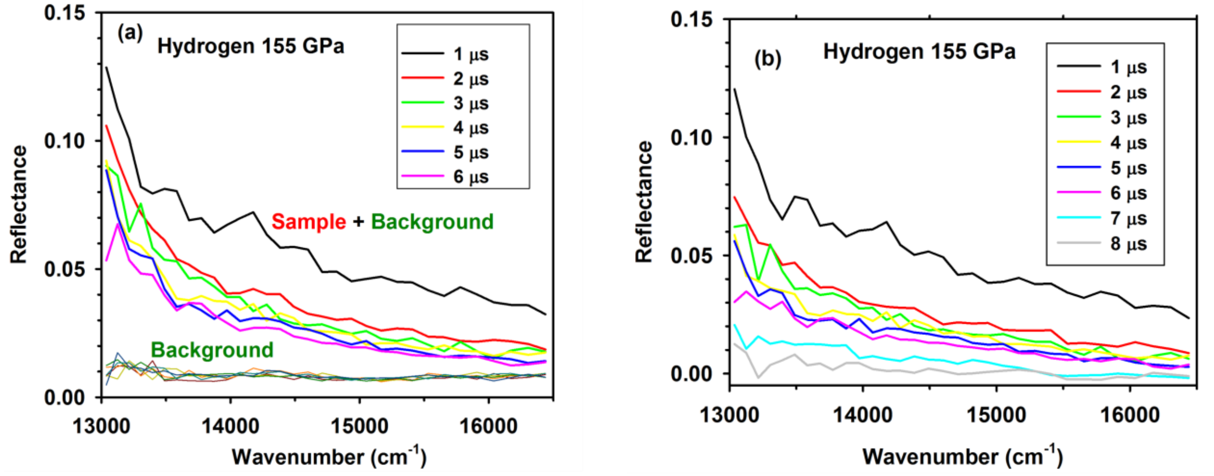

Figure S6. Reflectance spectra of hydrogen at 155 GPa. The heating pulse arrives near 0th microsecond. The spectra shown at the different times after the arrival of the heating pulse (see the legend). Left panel (a): raw data of the reflectance measured using SC pulses before the heating (background); Right (b): the background is subtracted.

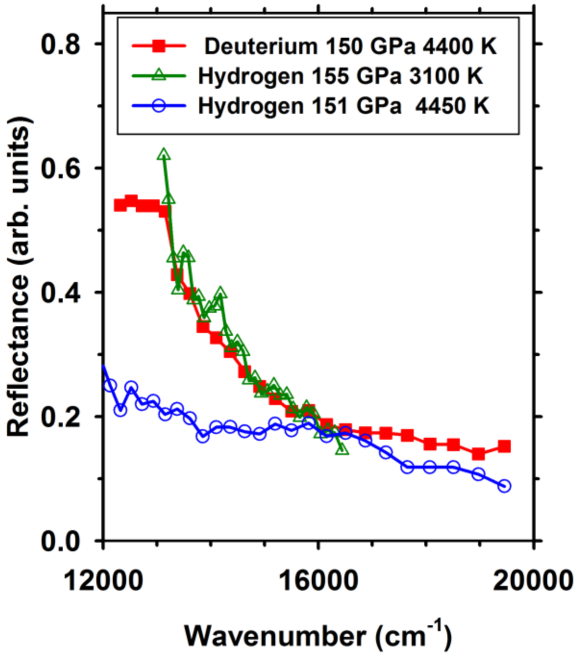

Figure S7. Reflectance spectra of hydrogens in different experiments. The spectra are scaled to roughly match the reflectance values at about 15000-20000 cm^{-1}.

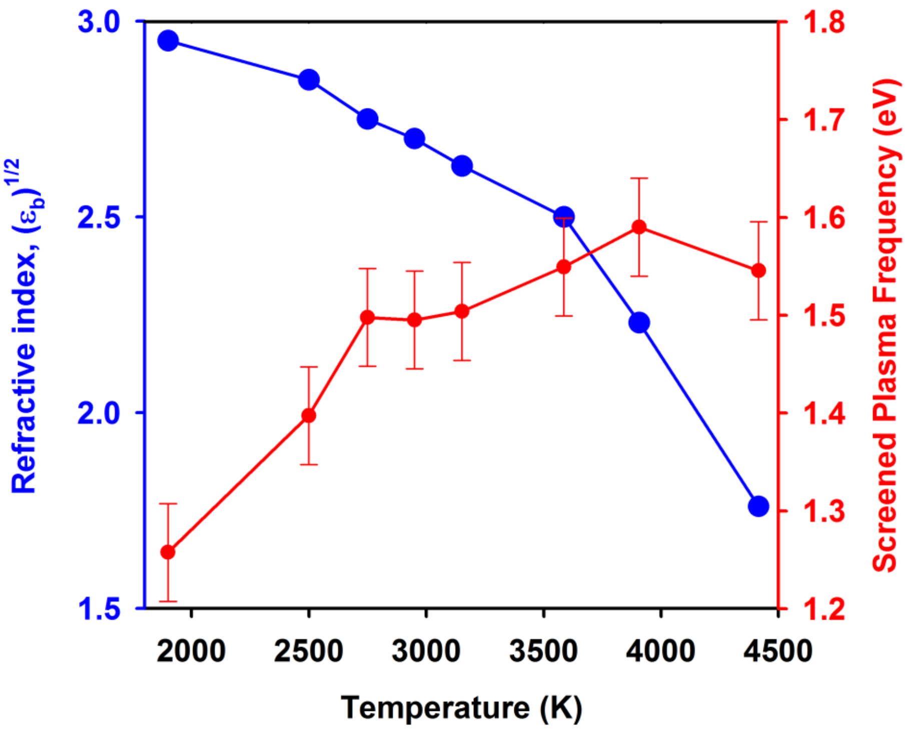

Figure S8. Parameters of the Drude model that have been used to fit the optical reflectance data of Fig. 3. The results are obtained in the same experiment on cooling down from the top temperature. Temperatures below 3000 K are determined via a linear extrapolations as a function of time.

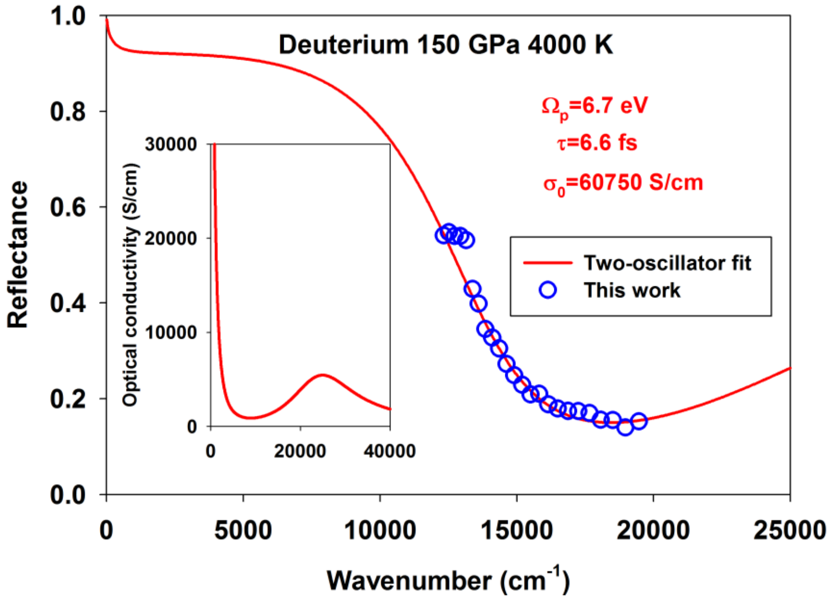

Figure S9. Reflectance spectrum of metallic deuterium fit using a model of two Lorentz oscillators.

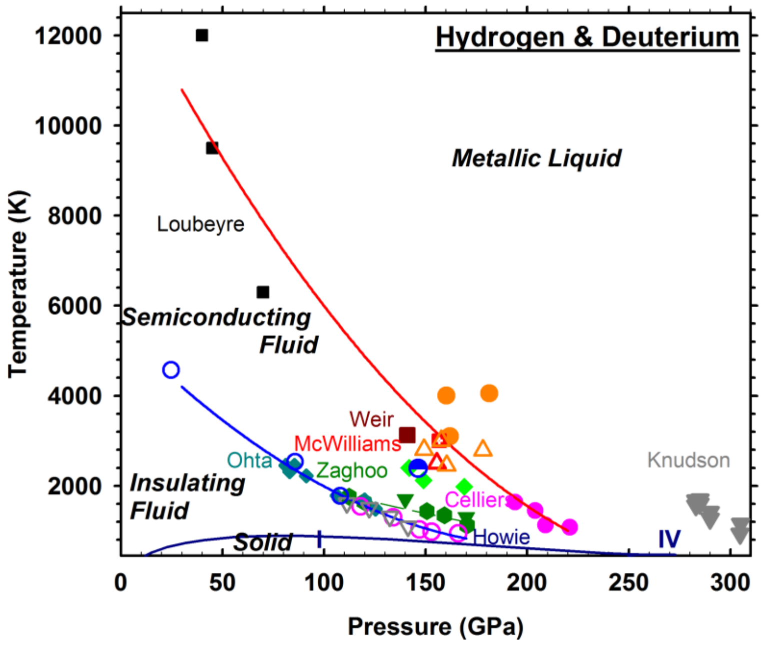

Figure S10. Phase diagram of hydrogen at extreme *P-T* conditions (in linear coordinates). Filled orange circles and filled crossed red square indicate conditions of the metallic state detected via optical reflectance in this study for hydrogen (H) and deuterium (D), respectively. Open orange and crossed red upward triangles correspond to P-T conditions where H and D reflectance respectively was lower than a few percent and our Drude analysis shows a sharp decline in the DC conductivity (Fig. 3). The uncertainty of temperature measurements is about 15% (1σ). Blue crosses are the conditions of absorbing H directly measured using a similar DAC technique as in this work [2]. Open and filled pink circles (gray triangles) are the results of gradual laser compression at NIF [6] (Z-machine [7]) corresponding to reaching the absorptive and reflecting D states, respectively. Solid brown square is the result of reverberating shock experiments detecting metallic H by electrical conductivity measurements [7, 8]. Open and solid dark blue diamonds show the P-T conditions of the volume discontinuity detected in the shock wave experiments of Ref. [9]. Filled black squares are IMT measured in single-shock experiments in precompressed samples; no major differences between D and H were indicated [10]. The results of DAC optical experiments reported as an abrupt insulator-metal transition are shown by dark green for H and light green for D [11-13]. Solid cyan diamonds are DAC experiments in H showing change in temperature vs. heating power dependence interpreted as phase transformation to a metal [14, 15]. Solid red and blue line through the data are the suggested phase boundaries for semiconducting and metallic hydrogen. The melting curve and solid state boundaries are from Ref. [4]. A thermal pressure of 2.5 GPa/1000 K is included[2].

References for SI reference citations

1. McWilliams, R. S.; Dalton, D. A.; Konôpková, Z.; Mahmood, M. F.; Goncharov, A. F., Opacity and conductivity measurements in noble gases at conditions of planetary and stellar interiors. *Proceedings of the National Academy of Sciences of the United States of America* **2015,** *112*, 7925-7930
2. McWilliams, R. S.; Dalton, D. A.; Mahmood, M. F.; Goncharov, A. F., Optical Properties of Fluid Hydrogen at the Transition to a Conducting State. *Phys Rev Lett* **2016,** *116* (25), 255501.
3. Montoya, J. A.; Goncharov, A. F., Finite element calculations of the time dependent thermal fluxes in the laser-heated diamond anvil cell. *Journal of Applied Physics* **2012,** *111* (11), 112617.
4. Howie, R. T.; Dalladay-Simpson, P.; Gregoryanz, E., Raman spectroscopy of hot hydrogen above 200 GPa. *Nat Mater* **2015,** *14* (5), 495-499.
5. Jiang, S.; Holtgrewe, N.; Lobanov, S. S.; Su, F.; Mahmood, M. F.; McWilliams, R. S.; Goncharov, A. F., Metallization and molecular dissociation of dense fluid nitrogen. *Nature Communications* **2018,** *9*, 2624.
6. Celliers, P. M.; Millot, M.; Brygoo, S.; McWilliams, R. S.; Fratanduono, D. E.; Rygg, J. R.; Goncharov, A. F.; Loubeyre, P.; Eggert, J. H.; Peterson, J. L.; Meezan, N. B.; Pape, S. L.; Collins, G. W.; Jeanloz, R.; Hemley, R. J., Insulator-metal transition in dense fluid deuterium. *Science* **2018,** *361*, 677–682.
7. Knudson, M. D.; Desjarlais, M. P.; Becker, A.; Lemke, R. W.; Cochrane, K. R.; Savage, M. E.; Bliss, D. E.; Mattsson, T. R.; Redmer, R., Direct observation of an abrupt insulator-to-metal transition in dense liquid deuterium. *Science* **2015,** *348* (6242), 1455-1460.
8. Weir, S. T.; Mitchell, A. C.; Nellis, W. J., Metallization of Fluid Molecular Hydrogen at 140 GPa (1.4 Mbar). *Phys Rev Lett* **1996,** *76* (11), 1860-1863.
9. Fortov, V. E.; Ilkaev, R. I.; Arinin, V. A.; Burtzev, V. V.; Golubev, V. A.; Iosilevskiy, I. L.; Khrustalev, V. V.; Mikhailov, A. L.; Mochalov, M. A.; Ternovoi, V. Y.; Zhernokletov, M. V., Phase Transition in a Strongly Nonideal Deuterium Plasma Generated by Quasi-Isentropical Compression at Megabar Pressures. *Phys Rev Lett* **2007,** *99* (18), 185001.
10. Loubeyre, P.; Brygoo, S.; Eggert, J.; Celliers, P. M.; Spaulding, D. K.; Rygg, J. R.; Boehly, T. R.; Collins, G. W.; Jeanloz, R., Extended data set for the equation of state of warm dense hydrogen isotopes. *Phys Rev B* **2012,** *86* (14), 144115.
11. Zaghoo, M.; Salamat, A.; Silvera, I. F., Evidence of a first-order phase transition to metallic hydrogen. *Phys Rev B* **2016,** *93* (15), 155128.
12. Zaghoo, M.; Silvera, I. F., Conductivity and dissociation in liquid metallic hydrogen and implications for planetary interiors. *Proceedings of the National Academy of Sciences* **2017,** *114* (45), 11873-11877.
13. Zaghoo, M.; Husband, R. J.; Silvera, I. F., Striking isotope effect on the metallization phase lines of liquid hydrogen and deuterium. *Phys Rev B* **2018,** *98* (10), 104102.
14. Ohta, K.; Ichimaru, K.; Einaga, M.; Kawaguchi, S.; Shimizu, K.; Matsuoka, T.; Hirao, N.; Ohishi, Y., Phase boundary of hot dense fluid hydrogen. *Scientific Reports* **2015,** *5*, 16560.
15. Dzyabura, V.; Zaghoo, M.; Silvera, I. F., Evidence of a liquid–liquid phase transition in hot dense hydrogen. *Proceedings of the National Academy of Sciences* **2013,** *110* (20), 8040-8044.
